# Habitat Preferences and Trophic Position of *Brachydiplax chalybea flavovittata* Ris, 1911 (Insecta: Odonata) Larvae in Youngsan River Wetlands of South Korea

**DOI:** 10.3390/insects11050273

**Published:** 2020-04-30

**Authors:** Jong-Yun Choi, Seong-Ki Kim, Jeong-Cheol Kim, Soon-Jik Kwon

**Affiliations:** 1 National Institute of Ecology, Seo-Cheon Gun 325-813, Chungcheongnam Province, Korea; skkim@nie.re.kr (S.-K.K.); jckim@nie.re.kr (J.-C.K.); 2 Institute for Ecological Resource, Seoul 02783, Korea; triopsidae@naver.com

**Keywords:** atmospheric temperature, distribution and diffusion, habitat heterogeneity, macrophytes, Odonata larvae, stable isotope analysis

## Abstract

In freshwater ecosystems, habitat heterogeneity supports high invertebrate density and diversity, and it contributes to the introduction and settlement of non-native species. In the present study, we identified the habitat preferences and trophic level of *Brachydiplax chalybea flavovittata* larvae, which were distributed in four of the 17 wetlands we examined in the Yeongsan River basin, South Korea. Larval density varied across four microhabitat types: open water area, and microhabitats dominated by *Myriophyllum aquaticum*, *Paspalum distichum*, and *Zizania latifolia*. Microhabitats dominated by *M. aquaticum* had the highest larval density, followed by those dominated by *P. distichum*. The larvae were more prevalent in silt sediments than in plant debris or sand. Stable isotope analysis showed that *B. chalybea flavovittata* is likely to consume, as a food source, other species of Odonata larvae. We conclude that successful settlement of *B. chalybea flavovittata* can be attributed to their habitat preferences. As temperature increases due to climate change, the likelihood of *B. chalybea flavovittata* spreading throughout South Korea increases. We, therefore, recommend continued monitoring of the spread and ecological impacts of *B. chalybea flavovittata*.

## 1. Introduction

Invertebrate communities play an important role in the functioning of freshwater wetland ecosystems. Aquatic invertebrates occupy an intermediate level in the freshwater food web, between phyto- and zooplankton and fish, and they are crucial for regulating food web dynamics [1]. Because spatiotemporal distribution patterns of invertebrates directly influence the population growth and fecundity of other major components of wetland food webs, these organisms have a strong impact on ecosystem health. Given their intermediate trophic position, the aquatic invertebrates require habitat conditions that not only supply sufficient food resources, but also provide refuge from predators. Empirical studies suggested that interactions such as competition and predation can induce shifts in habitat preferences and spatial distribution within invertebrate communities [2]. For example, areas populated by emergent macrophytes, such as *Phragmites communis* Trin. and *Typha orientalis* Presl., are unable to support high densities of cladocerans and rotifers owing to the relatively simple habitat structure they provide [3,4]. However, these macrophytic species are suitable habitats for damselflies, which move by crawling along solid stems [5]. Such niche partitioning allows species to coexist and fosters species diversity [6,7,8,9]. Therefore, the distribution patterns and habitat preferences of invertebrates should be identified in order to elucidate determinants of community structure.

Habitat heterogeneity provides numerous niches by increasing the diversity of ways in which organisms can exploit environmental resources [10]. Various microhabitats can support a wide diversity of invertebrates depending on the differential fitness among species in a heterogeneous space. The heterogeneity of microhabitats can be characterized not only by abiotic factors, such as water depth, wave action, turbulence, water temperature, and bottom substrates, but also by biotic structures [11,12], such as trees, woody debris, and composition and abundance of macrophytic communities [13,14]. Most wetland studies focused on the spatial distribution of invertebrates in heterogeneous habitat structures created by aquatic macrophytes that alter microhabitat complexity, as well as physical conditions, consequently affecting the abiotic and biotic characteristics of the ecosystem [15,16]. The leaves and stems of submerged macrophytes are more heterogeneous in structure than those of other macrophytic forms (e.g., emergent, free-floating, and floating-leaved) and, therefore, they increase the physical habitat complexity of their aquatic environment [17]. Field observations and experimental investigations confirmed the occurrence of high invertebrate densities in the presence of submerged macrophytes [18,19]. In addition, free-floating or floating-leaved macrophytes were also reported to fulfil important structuring functions in wetland systems [20]. Freshwater wetland ecosystems provide heterogeneous microhabitats with diverse structural complexity because of the mosaic of different habitats [21]. Thus, wetlands are able to support the settlement and population growth of various animal species.

In freshwater ecosystems, the geographical range extension and successful settlement of exotic species occurs within a stable habitat environment. In general, the migration of exotic species is explained by the effects of climate change, such as increased temperatures, or as an invasion through national or regional exchanges; however, exotic species introduced into ecosystems with limited ranges, such as wetlands, are closely related to habitat preferences. For example, *Lepomis macrochirus* spread throughout South Korean freshwater ecosystems because they find food source and refuge from predators in habitats with abundant macrophytes [22,23]. Another example is the African catfish (*Clarias gariepinus* Burchell, 1822), a species introduced into Brazil, which reduced fish species diversity by using native fishes as a food source [24]. The distribution and settlement of exotic species changes the interactions between other organisms; it requires adaptation on the part of native species and creates new ecological relationships. This can lead to the decline, extinction, or emigration of native species, or it may induce migration to other habitats, which can compromise ecological health by reducing biodiversity. Therefore, effective management and conservation of wetlands requires an understanding of the habitat preferences of introduced species and their relationships with native species.

Odonates (Insecta: Odonata) are important components of the freshwater invertebrate community, and they are essential for characterizing and assessing the land/water interface, as well as structural habitat heterogeneity and hydrological features of aquatic systems. They are suitable for use as indicator species, because their distribution, abundance, and diversity respond to environmental changes in temperature, pollution, and habitat structure [25,26]. Because their growth rate varies with temperature across latitude and altitude [27,28,29], the Korean Environment Ministry started to study climate change by monitoring several Odonata species as climate-sensitive biological indicator species (CBIS) [30]. Currently, the CBIS list includes three species: *Ceriagrion nipponicum* Asahina, 1967, *Ischnura senegalensis* Rambur, 1842, and *Brachydiplax chalybea flavovittata* Ris, 1911. For example, *B. chalybea flavovittata* is gradually moving northward toward the Yeongsan River or the Geum River ever since its first entry into Jeju Island was confirmed in 2010, and it is likely to spread widely, depending on changes in temperature. Although the overall distribution of this species was reported [31], its habitat preferences and interactions with other native species were not sufficiently studied. Furthermore, the presence of larvae—which would confirm successful settlement—was only confirmed in the area of Jeju Island, and not in the Yeongsan River basin.

In the present study, we investigated the distribution patterns and habitat preferences of *B. chalybea flavovittata* larvae in a riverine wetland located in the Yeongsan River basin, South Korea. The main purpose of this study was to describe the habitat preferences of *B. chalybea flavovittata* larvae and to evaluate their potential impact on native odonates in the Yeongsan River basin by describing their habitat and trophic niche requirements. We hypothesize that their ability to coexist with native species is a consequence of different niche requirements. To test this hypothesis, we investigated (i) the influence of hydrological characteristics and environmental variables on odonate larval distribution, (ii) the spatial distribution of *B. chalybea flavovittata* in different habitat types, and (iii) the trophic position of *B. chalybea flavovittata* and native Odonata larvae using stable isotope analysis. Based on our results, we discussed the settlement characteristics of *B. chalybea flavovittata* in South Korea and suggested new management strategies related to the promotion of biodiversity in freshwater wetlands.

## 2. Materials and Methods

### 2.1. Study Area

**Figure 1:**
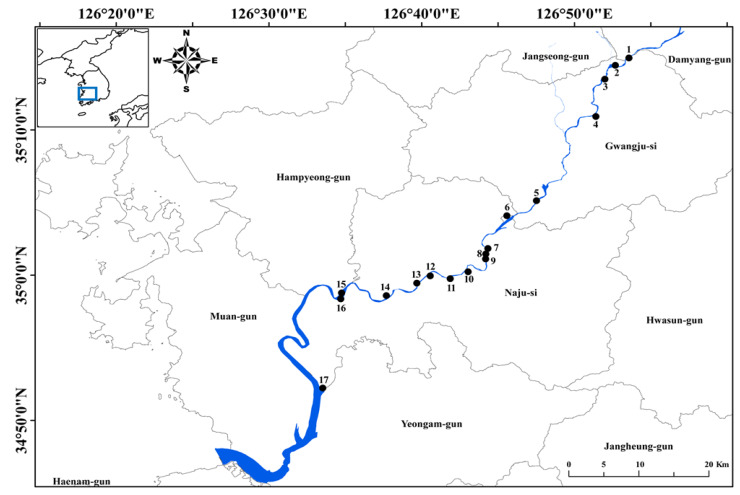
Map showing the 17 study sites located in southeastern South Korea. The study sites are indicated by solid circles (●). The small map in the upper left corner shows the Korean Peninsula.

South Korean freshwater ecosystems are temperate and have four distinct seasons. Mean annual rainfall is ca. 1150 mm, and more than 60% of annual rainfall occurs from June to early September [32,33]. The riverine wetlands included in this study are located in southwestern South Korea, around the mid and lower reaches of the Yeongsan River (Figure 1). Historically, there were numerous riverine wetlands within this Yeongsan River basin; however, large wetland areas vanished as a result of reorganization by the River Refurbishment Project in 2012. Agriculture is now the dominant land-use type in the basin, and non-point source pollution continuously influences these wetland ecosystems [34].

We investigated 17 riverine wetlands located in the Yeongsan River basin (Figure 1), which differ in basic morphological and limnological features (Table 1). Total water area ranges from 4000 to 350,000 m^2^ in each wetland; some of these water bodies are nearly circular, whereas others are long or oval. While the main water sources in most of these wetlands are streams, some rely on other water source types, such as rainfall, groundwater, and drainageways. Those that are primarily fed by drainageways have higher nutrient concentrations than those supplied by other types of water sources. Each wetland is characterized by a shallow littoral zone and a deeper limnetic zone, resulting in a clear separation of microhabitats. Macrophytes are abundant in and limited to the littoral zone. Study sites are highly covered by aquatic macrophytes, including *Phragmites australis*, *Paspalum distichum*, *Zizania latifolia*, *Spirodela polyrhiza*, *Salvinia natans*, *Trapa japonica*, *Ceratophyllum demersum*, and *Hydrilla verticillata*, in the period from spring (May) to autumn (November).

### 2.2. Monitoring Strategy

We monitored the study sites from May to June, before the summer monsoons and typhoons, in order to avoid flooding disturbance [35] and to obtain data under stable conditions. We established 3–5 sampling areas in the littoral zone at each site. At each sampling area, three quadrats (1 m × 1 m) were used to measure environmental variables and Odonata density.

Water temperature, percentage saturation of dissolved oxygen (DO), pH, conductivity, turbidity, chlorophyll a (Chl a), total nitrate (TN), and total phosphorus (TP) were measured in quadrats in each wetland. We used a DO meter (model 58; YSI Inc., Yellow Springs, OH, USA) to determine water temperature and DO; conductivity and pH were recorded using a conductivity meter (model 152; Fisher Scientific, Hampton, NH, USA) and an Orion 250A pH meter (Orion Research Inc., Boston, MA, USA), respectively. Water from a depth of 0.5 m to the surface was sampled using a 2-L column sampler. In order to determine Chl a concentration, water samples were filtered through 0.45-µm mixed cellulose ester membrane filters (A045A047A; Advantech Co. Ltd., Taipei, Taiwan). The filtered membranes were placed in cold 90% acetone, in darkness, at 20 °C for 4 h. To improve extraction, the cells were disintegrated for 2 min in an ultrasonic bath. To remove cell debris and filter particles, the pigment extract was centrifuged at 5000 rpm for 5–10 min. The extinction coefficient was estimated at 600 and 750 nm using a spectrophotometer (Japan Fantec Research Institute, Shizuoka, Japan), with the sample placed in a 1-cm glass cuvette [36]. The concentration of Chl a was estimated using the following formula:

Chl a = 11,403 × (A_600_ − A_750_) × V_a_ × V_b_^−1^, (1)

where V_a_ is the extract volume (mL) and V_b_ is the sample volume (mL). We also determined TN and TP spectrophotometrically, based on the method described in Wetzel and Likens [36].

In each quadrat, Odonata larvae collections were conducted for approximately 30 to 40 min using a stainless-steel sampler (30 cm width, 600 μm mesh). Based on habitat characteristics of odonate assemblages, we collected as many individuals as possible by sweeping over the sediment surface and over the leaves and stems of aquatic macrophytes. The sampling protocol was the same for all wetlands, and the three quadrats represented habitats characteristics within the littoral area. The collected odonate assemblages and organic material, including plant debris, were preserved in 10% formaldehyde. In the laboratory, each sample was washed through a 600-μm mesh sieve, and leaves, stems, and other debris were removed. The resulting material was preserved in 80% ethanol. Individual insects were separated and identified to species level according to Yoon [37], and Kawai and Tanida [38]. We established a species list of Odonata larvae for each wetland, and we compared the density of *B. chalybea flavovittata* larvae with that of other odonate species. After larval collection, aquatic macrophytes were collected in order to estimate their dry weight. Only the submerged parts of the macrophytes were used for the measurement of dry weight; the above-water stalks were removed from emergent macrophytes, and the above-water organs, such as flowers, were removed from free-floating and floating-leaved plants. The remaining plant masses were used to obtain the dry weight estimates (gram dry weight, gdw). This sampling strategy was also applied to the floating-leaved species. The collected macrophyte samples were dried at 60 °C for 48 h and weighed using an electronic microbalance (Mettler, AE 240, Switzerland).

In order to better understand the spatial distribution of *B. chalybea flavovittata* larvae with respect to different microhabitat characteristics, we conducted additional collections of these larvae in three wetlands (sites 1, 5, and 14) where they were abundant. We identified four different microhabitat types based on the heterogeneity of the macrophytic composition in each wetland: (1) open water area (without macrophytes), (2) area covered by *Myriophyllum aquaticum*, (3) area covered by *Paspalum distichum*, and (4) area covered by *Zizania latifolia*. Overall, we found very few aquatic free-floating or floating-leaved macrophytes. Although site 2 supported a moderate density of *B. chalybea flavovittata* larvae, it was excluded from additional investigations because of its relatively simple habitat structure (mostly covered by *P. distichum*). At each site, 80 randomly selected sampling points were surveyed from September to October. The quadrats (1 m × 1 m) were established at each sampling point for monitoring. We assigned 20 sampling points for each type of microhabitat. Moreover, we investigated the sediment types at 80 sampling points, and we compared the density of *B. chalybea flavovittata* larvae for each sediment type. We found three different sediment types in each wetland: (1) silt, (2) plant residue, and (3) sand. The “plant residue” means a sediment type in which the leaves or stems of aquatic plants are not decomposed or less decomposed. At each sampling point, water depth was measured with an echosounder (Simrad EK-500), and aquatic macrophyte biomass was collected, dried in the lab at 60 °C for two days, and weighed.

### 2.3. Stable Isotope Analysis

In order to compare the trophic levels of *B. chalybea flavovittata* and native Odonata species using stable isotope analysis, we collected five species of Odonata larvae, including *B. chalybea flavovittata*, at three wetlands (sites 1, 5, and 14) in which *B. chalybea flavovittata* larvae were abundant. The four other selected Odonata species (*Paracercion calamorum*, *Ischnura asiatica*, *Ceriagrion melanurum*, and *Sympetrum eroticum*) were the most dominant species in the spring survey. We captured as many individual Odonata larvae as possible in order to fulfill the minimum dry weight requirement for stable isotope analysis of at least 1.0 mg per sample.

The larvae samples were rinsed with deionized distilled water to remove the acid. All samples were freeze-dried and then ground with a mortar and pestle. All powdered samples were frozen (−70 °C) until the analysis. Nitrogen isotope ratios were determined using continuous-flow isotope mass spectrometry (CF-IRMS, model-ISOPRIME 100; Micromass Isoprime, GV Instruments Ltd., Manchester, UK). Prior to the analysis, the samples were placed in a sealed CF-IRMS overnight, with a 99.999% He flow of a few mL/min. Instrument linearity (dependence of δ^13^C and δ^15^N on signal amplitude at the collectors) was tested daily and confirmed to be <0.03‰/nA over the range of 1–10 nA. We loaded 100 ± 10 μg silver-encapsulated cellulose samples (no carbon was added to the samples inside the capsules), producing a signal of approximately 4–6 nA at the collectors, in a 99-position zero-blank CF-IRMS, and converted to a mixture of carbon monoxide, carbon dioxide, water, and hydrogen gases over glassy carbon chips in a quartz tube at 1080 °C, within a stream of 99.999% carrier He flowing at 110 mL/min. The data were expressed as the relative per mil (‰) difference between the sample and the conventional standards of Pee Dee Belemnite carbonate for carbon and atmospheric N_2_ for nitrogen, according to the following equation:

δ X (‰) = [(R_sample_/R_standard_) − 1] × 1000, (2)

where X is ^15^N and R is the ^15^N:^14^N ratio. A secondary standard of known relationship to the international standard was used as a reference material. The standard deviations δ^15^N for 20 replicate analyses of the peptone standard (δ^15^N = 7.0 ‰, Merck) were ±0.2 (‰).

### 2.4. Data Analysis

We used non-metric multidimensional scaling (NMDS) to examine Odonata distribution patterns according to environmental variations in 17 wetlands. The NMDS ordination plots were generated based on Euclidean distance, and goodness of fit was assessed in terms of loss of stress. Each variation was log-transformed after being assessed for normality with the Shapiro–Wilk test. The stress value for the two-dimensional solution was 0.132, which is lower than the generally accepted maximum stress value of <0.2 [39]. The significance of the fitted vectors was assessed using 3000 permutations, with *p* < 0.05 considered significant. NMDS ordination was conducted with the R package “vegan” (version 2.5-3 [40]).

We also used regression analysis to assess the influence of water depth and macrophyte biomass on the density of *B. chalybea flavovittata* larvae in each wetland. We tested linear, exponential, inverse, power, and logistic functions in order to determine the equation generating the best curve fit. The curve-fitting regression equation that returned the highest determination coefficient was selected to explain the observed relationships.

Furthermore, one-way ANOVA was used to examine the effects of microhabitat type and soil type on the mean density of *B. chalybea flavovittata* larvae, and differences in mean N values among the five odonate species. Tukey’s test was used for additional post hoc comparison analysis to determine which differences were statistically significant. 

All statistical analyses, including ANOVA, stepwise multiple regression, and species diversity analysis, were conducted using SPSS ver. 20 (released 2011; IBM SPSS Statistics for Windows, Version 20.0. Armonk, NY: IBM Corp.). Differences and relationships were considered significant at *p* < 0.05.

## 3. Results

### 3.1. Environmental Variables and Odonata Larvae Distribution

We found relatively little difference among the environmental variables of the study sites (Table 2). Although some study sites had exceptionally high or low values, the coefficients of variation (CV; standard deviation/mean × 100%) were lower than 100% in all study sites. The cover rate of macrophytes and DO had the highest CV, but the variation was only ca. 31.2% and 30.4%, respectively. The regression analysis between DO and cover rate of macrophytes indicated a positive relationship between these parameters (*r*^2^ = 0.68, *p* < 0.05). The DO values decreased as the cover rate of macrophytes increased. No statistical differences were found between other environmental variables.

**Figure 2:**
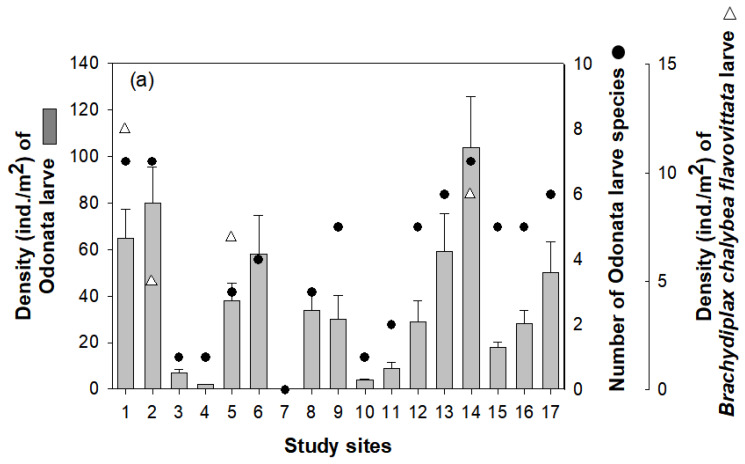
Density and species number (black closed circles) of Odonata larvae in 17 riverine wetlands. Densities of *Brachydiplax chalybea flavovittata* larvae are indicated by open triangles.

**Figure 3:**
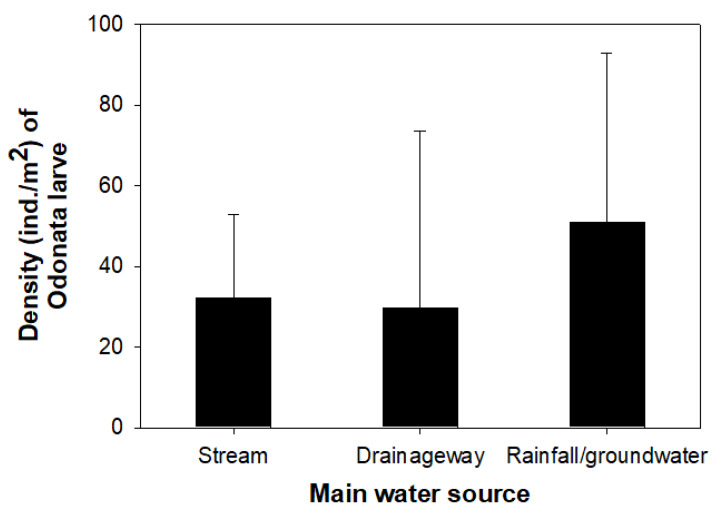
Density of Odonata larvae according to three main water sources.

A total of 15 Odonata species were identified from 17 wetlands. *Ceriagrion melanurum* was the most dominant species in study sites (relative richness: 28.1%), followed by *S. eroticum* (14.1%), *P. calamorum* (13.6%), and *I. asiatica* (12.2%). The relative richness of other Odonata species was less than 6%. The density and species number of odonate larvae differed among study sites (Figure 2). With over 100 ind./m^2^, site 14 supported the highest density of odonate larvae, followed by densities of more than 50 ind./m^2^ in sites 1, 2, 12, and 16. Furthermore, their density was abundant in wetlands with “rainfall/groundwater” among main water source types (Figure 3). Although, statistical significance was not verified, the wetlands with “stream” or “drainageway” supported relatively lower odonate larvae density than wetlands with “rainfall/groundwater”. The results of the NMDS indicated that the measured environmental variables did not influence the density of odonate larvae. Only four out of 17 wetlands were found to support *B. chalybea flavovittata* larvae (sites 1, 2, 5, and 14), and their densities ranged from 5 to 12 ind./m^2^.

### 3.2. Distribution of *Brachydiplax chalybea flavovittata* Larvae in Different Microhabitat Types

**Figure 4:**
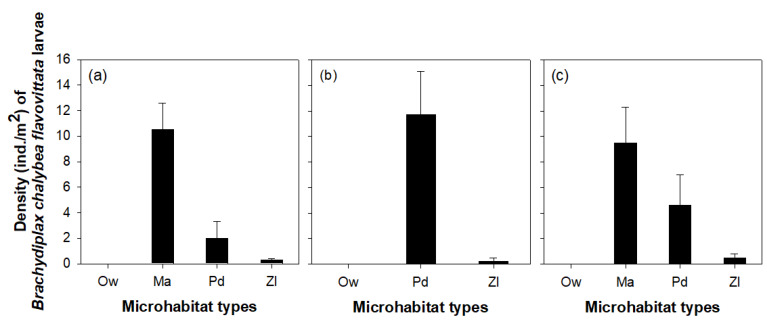
Density of *Brachydiplax chalybea flavovittata* larvae according to microhabitat type: Ow, open water area; Ma, area covered by *Myriophyllum aquaticum*; Pd, area covered by *Paspalum distichum*; Zl, area covered by *Zizania latifolia*. (**a**) site 1, (**b**) site 5, and (**c**) site 14.

The density of *B. chalybea flavovittata* larvae clearly differed among microhabitat types (one-way ANOVA, *p* < 0.05; Figure 4). We observed similar distribution patterns of *B. chalybea flavovittata* larvae in three wetlands where they were abundant (sites 1, 5, and 14). In sites 1 and 14, the area covered by *M. aquaticum* (Ma) supported the largest density of *B. chalybea flavovittata* larvae (site 1, 10.5 ± 2.1 ind./m^2^; site 14, 9.5 ± 2.8 ind./m^2^), followed by the area covered by *P. distichum* (Pd; site 1, 2.0 ± 1.3 ind./m^2^; site 14, 4.6 ± 2.4 ind./m^2^). In the absence of Ma, the larvae were most concentrated in areas covered by Pd (site 5, 11.7 ± 3.4 ind./m^2^; Figure 4b). The lowest larval density was observed in areas covered by *Z. latifolia* (Zl), and no larvae were found in open water areas.

**Figure 5:**
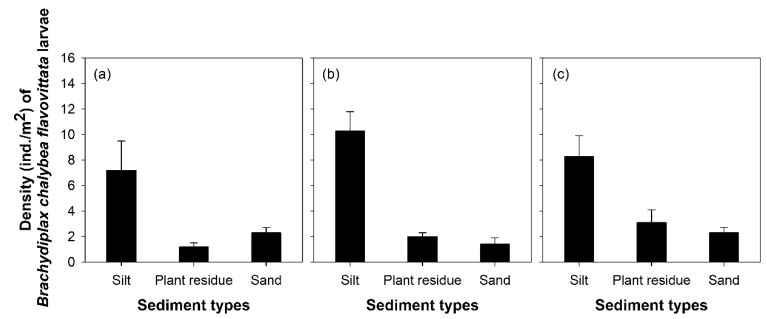
Density of *Brachydiplax chalybea flavovittata* larvae in three sediment types. (**a**) site 1, (**b**) site 5, and (**c**) site 14.

The density of *B. chalybea flavovittata* larvae also differed among the three sediment types (one-way ANOVA, *p* < 0.05; Figure 5). These larvae were more abundant in silt (site 1, 7.2 ± 2.3 ind./m^2^; site 5, 10.3 ± 1.5 ind./m^2^; site 14, 8.3 ± 1.6 ind./m^2^) than in other substrate types. Plant residue and sand substrates supported different densities of *B. chalybea flavovittata* larvae in different study sites. Site 1 supported a relatively high density of these larvae in sandy substrates (Figure 5a), whereas the larvae were more abundant in plant residue substrates of sites 5 and 14. The differences in larval densities were lower between plant residue and sand than those between these substrates and silt.

**Figure 6:**
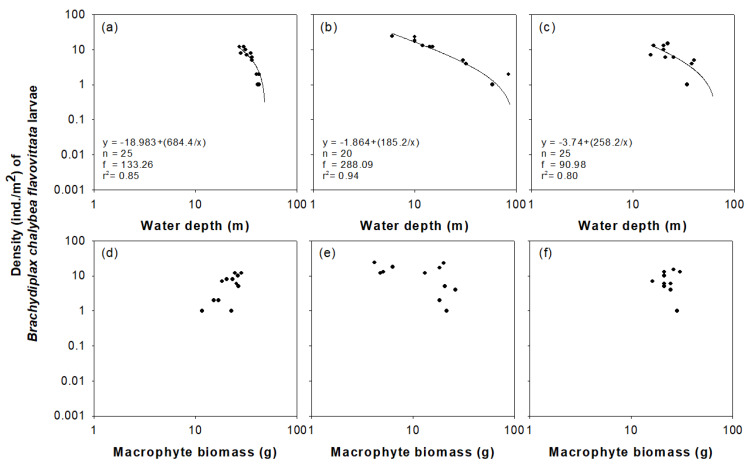
The relationships between the density of *Brachydiplax chalybea flavovittata* larvae and water depth at sites 1, 5, and 14 (**a**–**c**), and with macrophyte biomass at sites 1, 5, and 14 (**d**–**f**).

Regression analysis (Figure 6) showed a clear relationship between two environmental variables (water depth and macrophyte biomass) and density of *B. chalybea flavovittata* larvae. A power function generated the highest coefficient of determination between water depth and larval density. Density decreased with increasing water depth in all three wetlands (sites 1, 5, and 14; Figure 6a–c), and did not show any statistically significant correlation with macrophyte biomass (Figure 6d–f).

### 3.3. Stable Nitrogen Isotope Analysis of Odonata Larvae

**Figure 7:**
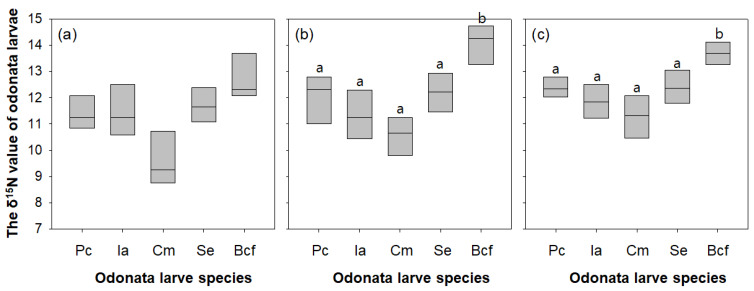
The δ^15^N value of each species of Odonata larvae in three wetlands (site 1, 5, and 14). Pc, *Paracercion calamorum*; Ia, *Ischnura asiatica*; Cm, *Ceriagrion melanurum*; Se, *Sympetrum eroticum*; Bef, *Brachydiplax chalybea flavovittata*. The three graphs (**a**–**c**) represent different three study sites (from the left, site 1, 5, and 14). Means labeled with a different letter indicate statistical subgroups defined by the post hoc test (Tukey honestly significant difference (HSD), *p* = 0.05).

The δ^15^N value differed significantly among the five sampled Odonata larvae species (one-way ANOVA, *p* < 0.05; Figure 7b,c). The δ^15^N values of larvae collected at site 1 showed no statistical differences, and they displayed a range similar range to that of the other sites (Figure 7a). The five species were clearly divided into two subgroups (a group with four dominant species, and b group with *B. chalybea flavovittata*) by the post hoc Tukey test. The δ^15^N value of *B. chalybea flavovittata* larvae ranged from 10.3‰ to 13.3‰ and was relatively heavier than that of other four species. The δ^15^N value of *C. melanurum* was the lightest (8.2‰ to 12.7‰), whereas those of the other three dominant Odonata species were similar. The δ^15^N value of the five investigated Odonata species showed a similar pattern among the three sampled wetlands.

## 4. Discussion

### 4.1. Influence of Environmental Variables on Odonata Larvae Distribution

Odonata communities were not strongly influenced by environmental variables or hydrological characteristics in any of the investigated wetlands. Aquatic organisms, such as fish and zooplankton, are known to be sensitive to chemical and physical factors such as dissolved oxygen, habitat structures, and water temperature, flow, and depth [41,42,43,44]. In contrast, invertebrates, including Odonata larvae, are less influenced by regional environmental characteristics, as they are relatively less mobile than aquatic organisms and spend most time occupying substrates (e.g., leaves and stem of plant, or sediment [45,46]). Moreover, while the high swimming ability of fish and the short life cycles of zooplankton enable them to respond rapidly to changes in wetland environments [47,48], odonate larvae, which are characterized by relatively slow movement and a long life cycle, have limited ability to respond to environmental fluctuations. In addition, Odonata adults are relatively mobile, able to disperse throughout multiple wetlands, and they are, therefore, capable of expanding the range of their larval habitats relatively quickly. Because of this, odonate larvae can have a wide distribution range throughout various lentic ecosystems, such as wetlands, reservoirs, and ponds. In the present study, we observed a moderate density of Odonata larvae in most of the study sites.

Although the environmental variables had little influence on Odonata larvae, we suggested that two factors contribute to population fluctuations. Firstly, Odonata larvae had relatively low density in wetlands supplied by drainageways. These wetlands are exposed to pollutants from nearby villages or farmland more frequently than wetlands that are primarily replenished by streams, rainfall, or groundwater. The resulting high nutrient loads can lead to eutrophication, which is harmful to invertebrate communities because of low DO levels and reduced productivity of important food sources, such as phyto- and zooplankton [49,50]. Moreover, wetlands primarily fed by streams, rainfall, or groundwater have a high abundance of littoral vegetation, whereas those fed primarily by drainageways often have artificial shorelines that lack a littoral zone, and they are, therefore, not suitable for the growth of aquatic macrophytes and, consequently, cannot support a high density of odonate larvae. Although our results showed little relationship between the density of Odonata larvae and the biomass of aquatic macrophytes, we observed that the larvae preferred areas that were moderately covered by aquatic macrophytes than open water area not covered by aquatic macrophytes.

Secondly, Odonata larvae were abundant in stable wetlands with little water flow. Water flow acts as a major source of disturbance for various aquatic organisms, including freshwater invertebrates, and it strongly affect species diversity and population growth [51,52]. In particular, the summer-concentrated rainfall of East Asian regions, including South Korea, negatively influences the autumn density of rotifers and cladocerans [33,53]. Previous studies suggested that a high abundance of aquatic macrophytes generally leads to habitat stabilization against physical disturbances, such as flow fluctuations and large amounts of summer rainfall. Ecosystems rich in aquatic macrophytes can consequently support a high density and diversity of invertebrates (e.g., rotifers [9,54]). In the present study, we observed high densities of Odonata larvae in wetlands where aquatic macrophytes were abundant.

### 4.2. Microhabitat Preference of *Brachydiplax chalybea flavovittata* Larvae

We observed variable densities of *B. chalybea flavovittata* larvae across four different microhabitat types, which indicated a clear habitat preference of the larvae. In general, Korean wetlands provide a suitable environment for the growth of various aquatic macrophytes, which can create highly heterogeneous habitats. Such habitat heterogeneity can induce stable settlement of exotic species, such as *B. chalybea flavovittata*, because it can support various organisms with different microhabitat preferences. The larvae of *B. chalybea flavovittata* had a greater preference for area covered by *M. aquaticum* than for those covered by other aquatic macrophyte species. *Myriophyllum aquaticum* provides a relatively more complex habitat structure than other macrophytes species because it is very densely distributed in the water and, therefore, it provides a suitable habitat for diverse species of Odonata larvae, including those of *B. chalybea flavovittata*. The greater preference for the areas covered by *M. aquaticum* could be driven by an indirect positive effect of the presence of more prey abundance and diversity. Interestingly, *B. chalybea flavovittata* larvae were concentrated in the areas dense with *P. distichum* in the absence of *M. aquaticum*, indicating that this is a viable alternative habitat for the larvae. Similarly, Cazzanelli et al. [55] suggested that free-floating macrophytes are important as they create microhabitats for invertebrates in water bodies, where submerged macrophytes are scarce. Choi et al. [56] and Sakuma et al. [57] also reported that some cladoceran species migrate from plant to plant according to the seasonal growth of aquatic macrophytes. This led us to consider that the spatial distribution of *B. chalybea flavovittata* larvae could depend on habitat heterogeneity and structure created by aquatic macrophytes in wetlands.

Although aquatic macrophytes provide habitat structure and are clearly influential on the density of *B. chalybea flavovittata* larvae, it is also important to determine the substrate types that are preferentially inhabited by *B. chalybea flavovittata* larvae. Unlike pelagic invertebrates, *B. chalybea flavovittata* larvae typically inhabit the substrate surface or interstices. Therefore, the observed variations in density depending on habitat characteristics associated with aquatic macrophytes are likely to be affected by sediment characteristics in each microhabitat type. We found that the *B. chalybea flavovittata* larvae were more abundant in silty substrates than in plant residue or sand substrates. Each of these sediment types has different organic composition. In an aquatic environment, the process of decomposition may be affected by many factors, including nutrients [58,59], temperature [60], pH [61], plant detritus availability, chemical composition, C:N:P ratio, microbiota metabolic activity, biomass, and diversity. The degree of decay of macrophytic leaves and stems can seasonally affect sediment composition in different microhabitat types [62]. Macrophytes with soft, perishable stems and leaves can quickly decay into soil components of relatively small size [63]. Accordingly, we found that areas with abundant *M. aquaticum* had silt substrates. Conversely, *P. distichum* and *Z. latifolia*, which have hard stems, generate large debris particles and coarse sediments because they decompose more slowly than plants with softer stems [64]. This sediment type is not suitable for Odonata larvae because of its low dissolved oxygen content and lack of food resources.

The high degree of preference of *B. chalybea flavovittata* larvae for a certain microhabitat type (i.e., *M. aquaticum*) and sediment type (i.e., silt) can explain why they are usually observed inhabiting shallow water depths. *Myriophyllum aquaticum* plants are mainly distributed in shallow waters and do not grow readily on silt sediment [65]. Some studies reported that *M. aquaticum* growth is possible even in areas with little water [66]. Furthermore, shallow water depth can alter competitive and trophic dynamics. In wetlands with relatively high water levels, Odonata larvae are vulnerable to predation by fish or competition with invertebrates that inhabit the euphotic and profundal zones. In particular, previous studies reported that *L. macrochirus* is widely distributed in South Korea and vigorously feeds on invertebrate prey, even in areas with a high abundance of aquatic macrophytes [23,67]. Wetlands or shallow water may support high abundance and species diversity of invertebrates that would otherwise be vulnerable to competition or predation.

Based on our findings, we concluded that the investigated Korean wetlands constituted a suitable habitat for *B. chalybea flavovittata* larvae. These wetlands are constantly supplied with nutrients such as nitrogen and phosphorus from surrounding agricultural land; this can potentially create various microhabitats by promoting the growth and development of aquatic macrophytes. These characteristics not only lead to a stable settlement of *B. chalybea flavovittata*, but also increase the likelihood that this species could spread widely throughout South Korea.

### 4.3. Impact of *Brachydiplax chalybea flavovittata* Settlement on Native Odonata Comunities

Stable nitrogen isotopes are frequently used to elucidate the interrelationships among freshwater organisms, allowing identification of not only the various prey items consumed by predators, but also the trophic levels of species [68]. Nitrogen isotope concentration in predators tends to be around 3–5‰, whereas that in prey species averages 3.0‰ ± 2.6‰, with a range of 0.5–9.2‰, [69]. Thus, the δ^15^N values of various organisms can indicate competition or prey–predator relationships. In the present study, we roughly estimated the trophic position of each investigated odonate species based on δ^15^N values. We found that the trophic position of *B. chalybea flavovittata* larvae was higher than that of other Odonata species larvae, indicating that *B. chalybea flavovittata* larvae are secondary or third consumers, consuming other animals. The composition of nitrogen isotopes in organic matter becomes heavier during the process of recycling through the ecosystem, and it reflects the trophic level of the organisms that consume them [70,71]. Empirical studies also suggested that Odonata larvae can utilize, as a food source, other Odonata larva species, as well as zooplankton such as cladocerans and copepods [72,73]. From these points, we assume that the high trophic position of *B. chalybea flavovittata* was attributed from consuming, as a food source, other Odonata species larvae. Interestingly, Pritchard [74] reported that Zygoptera formed a large part of the food of all Odonata larvae in freshwater ecosystems. However, we found that some wetlands where *B. chalybea flavovittata* larvae present were supported by the high abundance of other Odonata species larvae. This may be because the *B. chalybea flavovittata* larvae are in the early stages of settlement, or they may consume other food items excluding Odonata species larvae. Therefore, further analysis is needed on the utilization of food sources for the *B. chalybea flavovittata* larvae.

### 4.4. Geographical Extension and Settlement of *Brachydiplax chalybea flavovittata* Larvae in South Korea

The species *Brachydiplax chalybea flavovittata*, which was introduced into a wetland located in the Yeongsan River basin of South Korea, appears to have successfully naturalized here. The first report of *B. chalybea flavovittata* was in 2010 on Jeju Island; the species was later reported in the Yeongsan River and the Geum River basins in the period from 2014 to 2016, after which it gradually moved northward. However, it was not clear whether the Odonata in the Yeongsan River and Geum River basins settled inland, because only the adults were found. Our finding of larvae in the Yeongsan River basin indicates that this species settled in the inland area. It is known that *B. chalybea flavovittata* larvae can usually be found in hot and humid locations in India, Indonesia, and Thailand [75]; however, we found the larvae of this species in the period from May to November, within a temperature range that is much wider than that that tolerated by adults.

The settlement of *B. chalybea flavovittata* larvae in the Yeongsan River basin is closely related to the recent temperature rise in Korea [76]. This increase in average temperature induced the introduction and settlement of various non-native plants and animals, while populations of native species gradually declined or migrated. *Lycorma delicatula* White, 1845 and *Vespa velutina nigrithorax* Buysson, 1905, which were recently designated as invasive animal species in South Korea, are good examples of settlement as a consequence of climate change. Although these species were frequently introduced into South Korea in the past, it was only recently confirmed that a stable settlement was established [77,78]. Moreover, invasive plant species such as *Landoltia punctata* and *P. distichum* recently and rapidly began spreading in South Korea [79,80]. The settlement of *B. chalybea flavovittata* in the Yeongsan River basin is, therefore, an example of a continuous settlement pattern. As the average temperature of summer and winter is on a steady rise, it is highly likely that *B. chalybea flavovittata* will spread very widely in South Korea.

The distribution characteristics of freshwater ecosystems, along with rising temperatures in South Korea, also contribute to the diffusion of *B. chalybea flavovittata*. South Korean rainfall is mainly concentrated in summer and is relatively low in other seasons; thus, large numbers of wetlands or ponds were artificially created for continued use of water. The littoral area, with its shallow depth, can support a diverse invertebrate community because it is suitable for the growth of aquatic macrophytes. Moreover, the water flow in most sections of Korean rivers is restricted by small weirs; these areas now support various aquatic macrophytes. Considering these environmental conditions and the aforementioned habitat preferences of *B. chalybea flavovittata* larvae, we conclude that their diffusion in South Korea will be relatively rapid, assuming conducive climatic conditions.

The geographical range extension and settlement of exotic species commonly leads to new relationships and interactions between organisms within the freshwater food web. For example, *L. macrochirus* and *Micropterus salmoides*, which were introduced into South Korea in 1970, had a negative impact on native fish species because of their vigorous feeding activity and competition [81]. Although we did not find negative effects on the settlement of *B. chalybea flavovittata* larvae, the possibility cannot be ruled out based on the results of nitrogen isotope analysis. The trophic position of *B. chalybea flavovittata* larvae is such that this species does tend to interfere with native Odonata larvae, and ecological damage and disturbances by this species are present. As their settlement is still in its early stages, continuous monitoring of the extent of their spread and its ecological impact is needed.

## 5. Conclusions

We estimated that the successful settlement of *Brachydiplax chalybea flavovittata* in the Yeongsan River basin is closely related to low competition and the presence of their suitable microhabitat. Among the four microhabitat types associated with aquatic macrophytes, *B. chalybea flavovittata* preferred the microhabitats dominated by *Myriophyllum aquaticum*, and, among the three investigated sediment types, it preferred silt sediments. This species is likely to spread throughout South Korea because its suitable microhabitat type is very common in South Korea. However, we assume that *B. chalybea flavovittata* can consume other Odonata species larvae from stable isotope analysis. The relatively high trophic position of *B. chalybea flavovittata* was attributed from consuming other Odonata species larvae or utilizing food items with similar trophic position to Odonata larvae. We, therefore, recommend continued monitoring of the spread and ecological impacts of *B. chalybea flavovittata*.

## Figures and Tables

**Table 1:** Morphometric and limnological description of 17 investigated riverine wetlands. Fluctuation refers to the annual water level fluctuation (values > 1 m are regulated). Rainfall/ground, Rainfall/groundwater.

No.	Main Water Source	Altitude (m)	Area (m^2^)	Fluctuation (m)	Mean Depth (m)	Maximum Depth (m)	Mean Residence Time (year)
1	Stream	11.6	26,400	2.8	3.1	3.8	0.22
2	Drainageway	6.6	7800	<1	2.8	3.1	0.32
3	Stream	16.4	13,700	2.5	2.7	2.9	0.31
4	Stream	18.2	20,400	<1	4.1	4.6	0.16
5	Rainfall/ground	27.4	17,600	<1	2.4	2.7	0.36
6	Rainfall/ground	23.2	6700	<1	0.8	1.1	0.21
7	Drainageway	14.5	31,600	<1	1.6	2.0	0.12
8	Stream	12.8	13,600	1.7	3.4	3.6	0.21
9	Stream	16.5	15,900	1.1	2.8	3.2	0.15
10	Rainfall/ground	9.2	22,600	<1	1.2	1.8	0.43
11	Drainageway	11.8	25,600	<1	0.7	1.6	0.41
12	Stream	20.7	4000	3.4	2.3	3.1	0.31
13	Stream	17.6	27,600	2.8	1.1	1.6	0.22
14	Rainfall/ground	26.7	350,000	<1	0.8	1.4	0.42
15	Stream	24.3	137,957	1.7	1.8	2.2	0.18
16	Stream	30.5	109,000	2.5	1.6	2.1	0.11
17	Stream	24.7	137,957	1.4	2.3	2.7	0.37

**Table 2:** Environmental variables in 17 riverine wetlands. WT, water temperature; DO, dissolved oxygen; Cond., conductivity; Chl a, chlorophyll a; TN, total nitrogen; TP, total phosphorus; MB, macrophyte biomass; SD, standard deviation; CV, coefficient of variation (%).

No.	WT (°C)	DO (%)	pH	Cond. (µS/cm)	Turbidity (NTU)	Chl a (µg/L)	TN (mg/L)	TP (µg/L)	MB (g)
1	21.3	58.3	8.1	235.3	10.6	16.3	1.3	13.4	12.3
2	20.9	21.3	7.6	312.4	6.4	8.2	1.8	16.3	32.2
3	19.2	46.3	7.4	289.3	12.3	10.6	1.1	11.3	16.2
4	20.5	22.3	7.8	321.6	8.3	6.3	1.1	14.2	28.3
5	20.8	32.6	7.2	314.2	10.3	13.4	0.7	15.7	21.2
6	21.4	28.3	8.3	226.2	11.3	10.3	1.3	12.8	26.3
7	20.3	23.2	8.0	284.3	15.2	12.4	1.5	16.7	31.2
8	19.7	36.2	7.9	273.2	5.8	8.2	1.4	14.2	23.5
9	21.6	27.2	8.1	257.3	7.3	10.3	1.2	16.2	28.1
10	20.1	23.1	8.4	305.1	10.2	13.4	1.0	11.3	25.3
11	22.3	31.1	7.3	274.3	9.2	10.3	1.5	17.2	16.3
12	21.2	36.2	7.8	246.7	8.4	10.8	1.4	12.4	13.8
13	21.8	41.8	8.0	338.2	10.3	13.7	1.0	11.3	8.3
14	21.2	25.1	7.6	374.2	12.9	12.4	1.3	15.2	26.2
15	22.1	37.1	7.9	312.2	12.4	15.7	0.7	14.8	18.2
16	19.4	31.2	7.2	285.3	12.5	16.3	0.9	12.7	22.8
17	20.3	26.7	8.2	241.0	10.2	12.3	1.6	14.3	26.4
Mean	20.8	32.2	7.8	287.7	10.2	11.8	1.2	14.1	22.2
SD	0.9	9.8	0.4	39.6	2.5	2.9	0.3	1.9	6.9
CV	4.4	30.4	4.8	13.8	24.3	24.2	24.9	13.8	31.2
